# N‐terminal pro‐B‐type natriuretic peptide and D‐dimer combined with left atrial diameter to predict the risk of ischemic stroke in nonvalvular atrial fibrillation

**DOI:** 10.1002/clc.23933

**Published:** 2022-10-08

**Authors:** Zican Shen, Dong Chen, Hao Cheng, Feng Tan, Jianwei Yan, Haiming Deng, Wei Fang, Sunan Wang, Jianbing Zhu

**Affiliations:** ^1^ Department of Cardiology The First Affiliated Hospital of Nanchang University Nanchang China; ^2^ Jiangxi Hypertension Research Institute Nanchang China; ^3^ Beijing Hospital of Traditional Chinese Medicine Capital Medical University Beijing China

**Keywords:** d‐dimer, ischemic stroke, LAD, nonvalvular atrial fibrillation, NT‐proBNP, predictive value

## Abstract

**Objectives:**

We aimed to explore the potential role of N‐terminal pro‐B‐type natriuretic peptide (NT‐proBNP), d‐dimer, and the echocardiographic parameter left atrial diameter (LAD) in identifying and predicting the occurrence of ischemic stroke (IS) in patients with nonvalvular atrial fibrillation (NVAF).

**Methods:**

We conducted a retrospective study of 445 patients with NVAF in the First Affiliated Hospital of Nanchang University. They were divided into the NVAF (309 cases) and NVAF with stroke (136 cases) groups according to whether acute ischemic stroke (AIS) occurred at admission. Multivariate logistic regression was used to analyze the odds ratio (OR) of NT‐proBNP, d‐dimer, and LAD for IS. The predictive value of NT‐proBNP, d‐dimer, and LAD in identifying the occurrence of IS in NVAF was determined by plotting the receiver operating characteristic (ROC) curves.

**Results:**

NT‐proBNP, d‐dimer, and LAD levels were significantly higher in the NVAF with stroke group than in the NVAF group (*p* < .05). NT‐ProBNP, d‐dimer, and LAD were independently associated with IS in NVAF patients (odds ratio [OR] = 1.12, 95% confidence interval [CI]: 1.08–1.16; OR = 1.87, 95% CI: 1.37–2.55; OR = 1.21, 95% CI: 1.13–1.28, *p* < .01). The optimal cutoff points for NT‐ProBNP, d‐dimer, and LAD levels to distinguish the NVAF group from the NVAF with stroke group were 715.0 pg/ml, 0.515 ng/ml, and 38.5 mm, respectively, with the area under the curve (AUC) being [0.801 (95% CI: 0.76–0.84); 0.770 (95% CI: 0.72–0.85); 0.752 (95% CI: 0.71–0.80), *p* < .01]. The combined score of NT‐proBNP, d‐dimer, and LAD improved the predictive efficacy of the single index, with an AUC of 0.846 (95% CI: 0.81–0.88, *p* < .01), sensitivity of 77.2%, and specificity of 76.4%.

**Conclusion:**

NT‐proBNP, d‐dimer, and the echocardiographic parameter LAD have outstanding value in predicting the risk of IS in patients with NVAF.

## 1 INTRODUCTION

Atrial fibrillation (AF) is currently the most common tachyarrhythmia in clinical practice, and its incidence is increasing every year.^1^ By the end of 2019, approximately 59.7 million patients had AF (including atrial flutter) worldwide.^2^ AF can lead to stroke, myocardial infarction, and heart failure, which greatly increase the risk of death in patients.^3^, ^4^, ^5^ Nonvalvular atrial fibrillation (NVAF) can be regarded as the main independent risk factor of ischemic stroke (IS).^6^ Compared with other causes of stroke, acute ischemic stroke (AIS) associated with NVAF not only has an acute onset, rapid progression, and serious neurological loss but also has a higher recurrence rate and mortality, which imposes a huge medical burden on society.^7^, ^8^, ^9^ Several AF‐related IS risk stratification tools have been established internationally, such as the CHADS2, CHA2DS2‐VASc, and ABC stroke scores.^10^ The CHA2DS2‐VASc score is currently the most widely used risk stratification tool in clinics and is used for the early identification of medium‐ and high‐risk patients.^11^

Previous studies^12^, ^13^, ^14^, ^15^, ^16^ have found that N‐terminal pro B‐type natriuretic peptide (NT‐proBNP), d‐dimer, and the cardiac structural index left atrial diameter (LAD) are strongly associated with the development of AF and could help refine the risk assessment of IS related to NVAF. PAULIN et al.^17^ combined three serum biomarkers, high‐sensitivity troponin (HsT), brain natriuretic peptide (BNP), and d‐dimer, with the general clinical characteristics of patients, and identified the potential value of hematological parameters in predicting the occurrence of IS in AF. Due to the complex process of thromboembolism in patients with NVAF, current research focuses on different markers. However, in view of their extensive clinical use, easy access, and promotion, they are still highly beneficial in guiding clinical work.

This study aimed to investigate the risk factors for NVAF‐related IS and analyze the potential value of NT‐proBNP, d‐dimer, and LAD in the early prediction of NVAF‐related IS. This provides a reliable basis for early prevention and treatment of NVAF‐related IS.

## 2 RESEARCH DESIGN AND METHODS

### 2.1 Study population

This retrospective study enrolled 445 patients (247 males and 198 females) diagnosed with NVAF at the First Affiliated Hospital of Nanchang University between June 2020 and July 2022, who met the inclusion criteria. According to whether AIS occurred at admission, there were 309 and 136 cases in the NVAF and NVAF with stroke groups.

**Figure 1:**
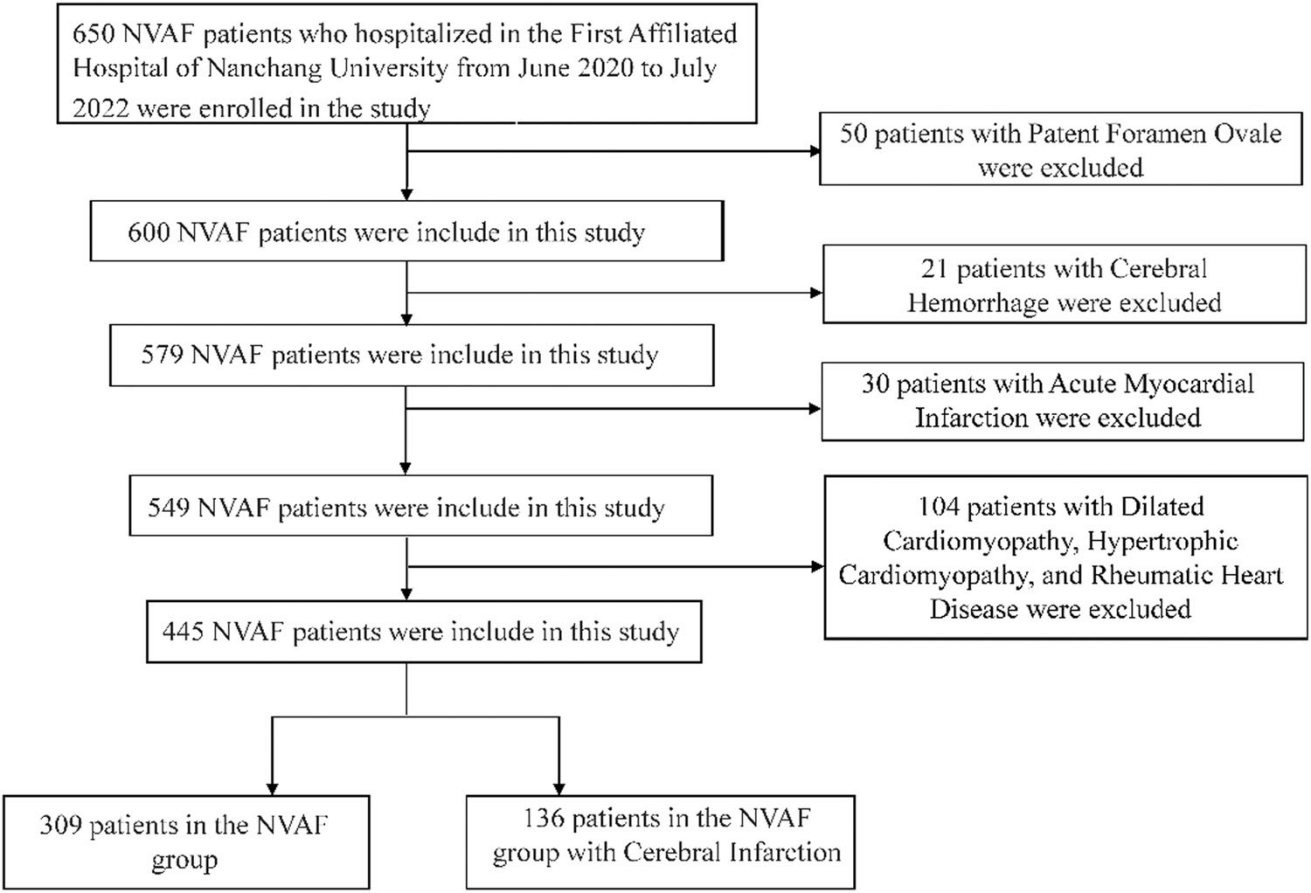
Flow diagram of processing for the selection of patients. NVAF, nonvalvular atrial fibrillation.

The inclusion criteria were as follows: (1) AF determined by past medical history, admission with 12‑lead electrocardiography (ECG) or 24‐h Holter monitoring, and nonvalvular diagnosis detected by cardiac ultrasound; (2) IS, including the current onset or previous history of IS, with a definite diagnosis of “AIS” by magnetic resonance imaging (MRI) on admission, but with other causes of IS excluded. The exclusion criteria were as follows: (1) cerebral hemorrhage, history of trauma surgery, or acute myocardial infarction combined with other types of arrhythmias within the last 3 months; (2) patent foramen ovale, valvular AF, rheumatic mitral valve disease, dilated cardiomyopathy, hypertrophic cardiac disease, and so forth; and (3) severe infection, severe liver and kidney dysfunction, thyroid disease, and malignant tumors. A flowchart of the entry process is shown in Figure 1.

### 2.2 Data collection

Patients who met the inclusion and exclusion criteria during hospitalization were analyzed by reviewing their medical records and collecting corresponding materials, including (1) basic demographic characteristics, such as sex, age, and body mass index (BMI); (2) medical or drug history, such as smoking, hypertension, diabetes mellitus, coronary heart disease (CHD), previous IS, antiplatelets, and anticoagulants; (3) serological indicators, such as NT‐proBNP, d‐dimer, fibrinogen, HsT, lipid quadruple, creatinine, uric acid, homocysteine, C‐reactive protein (CRP), and all test results obtained from the first venous blood test after admission and before treatment; (4) echocardiographic parameters, such as LAD, left ventricular end‐diastolic dimension (LVDd), and left ventricular ejection fraction (LVEF); and (5) CHA2DS2‐VASc score of all patients admitted.

### 2.3 Definition

NVAF was defined as AF without mechanical valves or moderate‐to‐severe mitral stenosis (usually resulting from rheumatism). Patients with NVAF included all three types of AF (paroxysmal AF, persistent AF, and permanent AF) with arbitrary durations.^18^, ^19^ IS was defined as neurological deficits caused by focal brain, spinal cord, or retinal infarction.^20^ BMI was defined as weight (kg) divided by the square of height (m) (kg/m^2^). Smoking is described as a total of ≥100 cigarettes in their lifetime, regardless of whether they have quit smoking now.^21^ History of hypertension refers to previous systolic blood pressure ≥ 140 mmHg and/or diastolic blood pressure ≥ 90 mmHg or use of antihypertensive drugs. A history of diabetes was defined as treatment with oral hypoglycemic agents or insulin, fasting glucose level ≥ 7.0 mmol/L, glycosylated hemoglobin ≥ 6.5%, or an oral glucose tolerance test showing 2‐h blood glucose ≥ 11.1 mmol/L. History of CHD was defined by previous episodes of angina pectoris or myocardial infarction, any positive cardiac stress test results, or coronary angiographic pathology signs.^22^ History of previous IS was defined as any neurological dysfunction event with or without sequelae.^22^

### 2.4 Risk stratification schemes for ischemic stroke

The CHA2DS2‐VASc score was calculated as congestive heart failure, hypertension, diabetes mellitus, vascular disease, age 65–74 years, and female sex; each of the above was scored as 1 point. A history of stroke, transient ischemic stroke, or age ≥75 years was scored as 2 points^23^ (Table S1).

### 2.5 Statistical analyses

Continuous variables with a normal distribution are described as mean ± standard deviation, while continuous variables with a non‐normal distribution are represented by quartiles. Categorical variables are summarized as frequencies and percentages. Categorical variables were compared using the Chi‐squared test or Fisher's exact test. The Student's *t*‐test for independent samples was used to compare two continuous variables with a normal distribution, and the Mann–Whitney *U* test was used to compare two continuous variables with a non‐normal distribution. Univariate and multivariate logistic regression analyses were used to explore independent risk factors for the development of IS in patients with NVAF. Receiver operating characteristic (ROC) curves were used to determine the predictive ability of CHA2DS2‐VASc, NT‐proBNP, d‐dimer, and LAD levels in patients with NVAF. Their optimal cutoff values, sensitivities, and specificities were calculated. Statistical significance was set at a two‐tailed *p*‐value < .05. Statistical analyses were performed using SPSS software (version 23.0; SPSS Inc.).

## 3 RESULTS

### 3.1 Demographic and clinical data

We analyzed the baseline data of 445 patients with NVAF, of which 247 (55.5%) were males and with a mean age of all patients of 71.3 ± 10.7 years. A total of 309 patients were included in the NVAF group and 136 patients were included in the NVAF with stroke group. The clinical characteristics of the two groups are shown in Table 1. The levels of homocysteine, cholesterol, triglycerides, low‐density lipoprotein (LDL), d‐dimer, fibrinogen, CRP, NT‐proBNP, HsT, LAD, and LVDd were all higher in the NVAF with stroke group (*p* < .05). In the CHA2DS2‐VASc score ≥ 2 subgroup segment, the within‐group percentage was higher in the NVAF with stroke group (96.3% vs. 77.0%, *p* < .01). In particular, the levels of NT‐proBNP, d‐dimer, and LAD in the NVAF with stroke group were significantly higher than those in the NVAF group (950.0 [665.3–1536.3] vs. 429.0 [89.6–725.3]; 1.2 [0.6–1.8] vs. 0.4 [0.2–0.8]; 43.1 [±4.7] vs. 37.6 [±6.2], *p* < .01). Patients in the NVAF with stroke group had lower LVEF (56.8 [±5.0] vs. 59.1 [±6.8], *p* < .01) and less anticoagulant use (19.9% vs. 31.1%) than those in the NVAF group.

**Table 1:** Baseline demographics and clinical data of included patients

Variable	ALL (*n* = 445)	NVAF (*n* = 309)	NVAF with stroke (*n* = 136)	*p* value
Age, years (mean ± SD)	71.3 (±10.7)	70.8 (±11.1)	72.5 (±9.8)	.136
Gender, male (*n*, %)	247 (55.5)	164 (53.1)	83 (61)	.120
BMI, kg/m^2^ (mean ± SD)	23.1 (±3.6)	23.2 (±3.8)	22.8 (±3.2)	.258
Smoking (*n*, %)	106 (23.8)	80 (25.9)	26 (19.1)	.122
Hypertension (*n*, %)	242 (54.4)	161 (52.1)	81 (59.6)	.146
Diabetes mellitus (*n*, %)	63 (14.2)	44 (14.2)	19 (14.0)	.940
CHD (*n*, %)	92 (20.7)	69 (22.3)	23 (16.9)	.194
Previous ischemic stroke (*n*, %)	78 (17.5)	47 (15.2)	31 (22.8)	.053
Previous medication (*n*, %)				
Antiplatelet	94 (21.1)	63 (20.4)	31 (22.8)	.567
Anticoagulant	123 (27.6)	96 (31.1)	27 (19.9)	.015
Admission biochemistry (median, IQR)				
Fasting blood glucose, mmol/L	5.4 (4.8–6.6)	5.5 (4.8–6.7)	5.2 (4.7–6.4)	.054
Homocysteine, μmol/L	13.0 (10.9–16.4)	12.8 (10.5–16.1)	13.9 (12.0–17.4)	.001
Cholesterol, mmol/L(mean ± SD)	4.0 (±0.9)	3.9 (±0.9)	4.2 (±0.9)	.001
Triglyceride, mmol/L(mean ± SD)	1.3 (±0.9)	1.4 (±1.0)	3.9 (±0.9)	.033
LDL, mmol/L (mean ± SD)	2.2 (±0.8)	2.2 (±0.8)	2.3 (±0.8)	.032
HDL, mmol/L (mean ± SD)	1.2 (±0.3)	1.2 (±0.3)	1.2 (±0.3)	.462
Creatinine, mmol/L	80.7 (67.5–99.0)	82.0 (68.7–99.4)	79.4 (65.4–98.7)	.366
Uric acid, mmol/L	357.1 (298.0–428.0)	358.0 (300.7–432.2)	343.5 (295.0–422.5)	.368
d‐dimer, ng/mL	0.5 (0.2–1.3)	0.4 (0.2–0.8)	1.2 (0.6–1.8)	<.01
Fibrinogen, g/L (mean ± SD)	3.0 (±0.8)	2.9 (±0.8)	3.1 (±0.9)	.011
CRP, mg/L	2.3 (0.8–6.5)	1.7 (0.8–4.7)	3.8 (1.9–10.9)	<.01
NT‐proBNP, pg/ml	617.0 (257.2–937.8)	429.0 (89.6–725.3)	950.0 (665.3–1536.3)	<.01
HsT, µg/ml	11.4 (6.7–20.5)	10.9 (5.5–18.9)	12.4 (9.0–23.1)	.001
Echocardiography parameters (mean ± SD)				
LAD, mm	39.3 (±6.3)	37.6 (±6.2)	43.1 (±4.7)	<.01
LVDd, mm	45.4 (±5.5)	44.1 (±5.3)	48.3 (±4.9)	<.01
LVEF, %	58.4 (±6.4)	59.1 (±6.8)	56.8 (±5.0)	<.01
CHA2DS2‐VASc score (*n*, %)				<.01
Score = 0	27 (6.1)	25 (8.1)	2 (1.5)	
Score = 1	49 (11.0)	46 (14.9)	3 (2.2)	
Score ≥ 2	369 (82.9)	238 (77.0)	131 (96.3)	

Abbreviations: BMI, body mass index; CHD, coronary heart disease; CRP, C‐reactive protein; HDL, high‐density lipoprotein; HsT, highly sensitive troponin; IQR, interquartile range; LAD, left atrial diameter; LDL, low‐density lipoprotein; LVDd, left ventricular end‐diastolic dimension; LVEF, left ventricular ejection fraction; NT‐proBNP, N‐terminal pro B‐type natriuretic peptide; NVAF, nonvalvular atrial fibrillation; SD, standard deviation.

### 3.2 Predictors of ischemic stroke

Transformed NT‐proBNP (100‐fold increments), d‐dimer, and echocardiographic parameter LAD were included in the logistic regression analysis (Table 2). After adjusting for demographics (sex, age, and BMI), clinical risk factors (smoking, hypertension, diabetes, CHD, previous IS, and history of oral antiplatelet and anticoagulant medications), and CHA2DS2‐VASc scores, we found that NT‐proBNP, d‐dimer, and LAD were independent risk factors for IS in patients with NVAF (odds ratio [OR] = 1.12, 95% confidence interval [CI]:1.08–1.16; OR = 1.87, 95% CI: 1.37–2.55; OR = 1.21, 95% CI: 1.13–1.28, *p* < .01).

**Table 2:** Univariate analysis and multivariate analysis model for NVAF with stroke

	Univariate analysis			Multivariate analysis^a^		
	OR	95％ CI	*p* value	OR	95％ CI	*p* value
Transformed NT‐proBNP	1.12	1.09–1.16	<.01	1.12	1.08–1.16	<.01
d‐dimer	2.21	1.70–2.87	<.01	1.87	1.37–2.55	<.01
LAD	1.19	1.14–1.24	<.01	1.21	1.13–1.28	<.01

Abbreviations: BMI, body mass index; CHD, coronary heart disease; CI, confidence interval; LAD, left atrial diameter; NT‐proBNP, N‐terminal pro B‐type natriuretic peptide; NVAF, nonvalvular atrial fibrillation; OR, odds ratio.

^a^ Adjusted for demographics (age, sex, and BMI), clinical risk factors (smoking, hypertension, diabetes mellitus, CHD, and history of stroke), medication history (antiplatelet and anticoagulant drugs), and admission CHA2DS2‐VASc score.

### 3.3 Predictive value of risk factors

**Figure 2:**
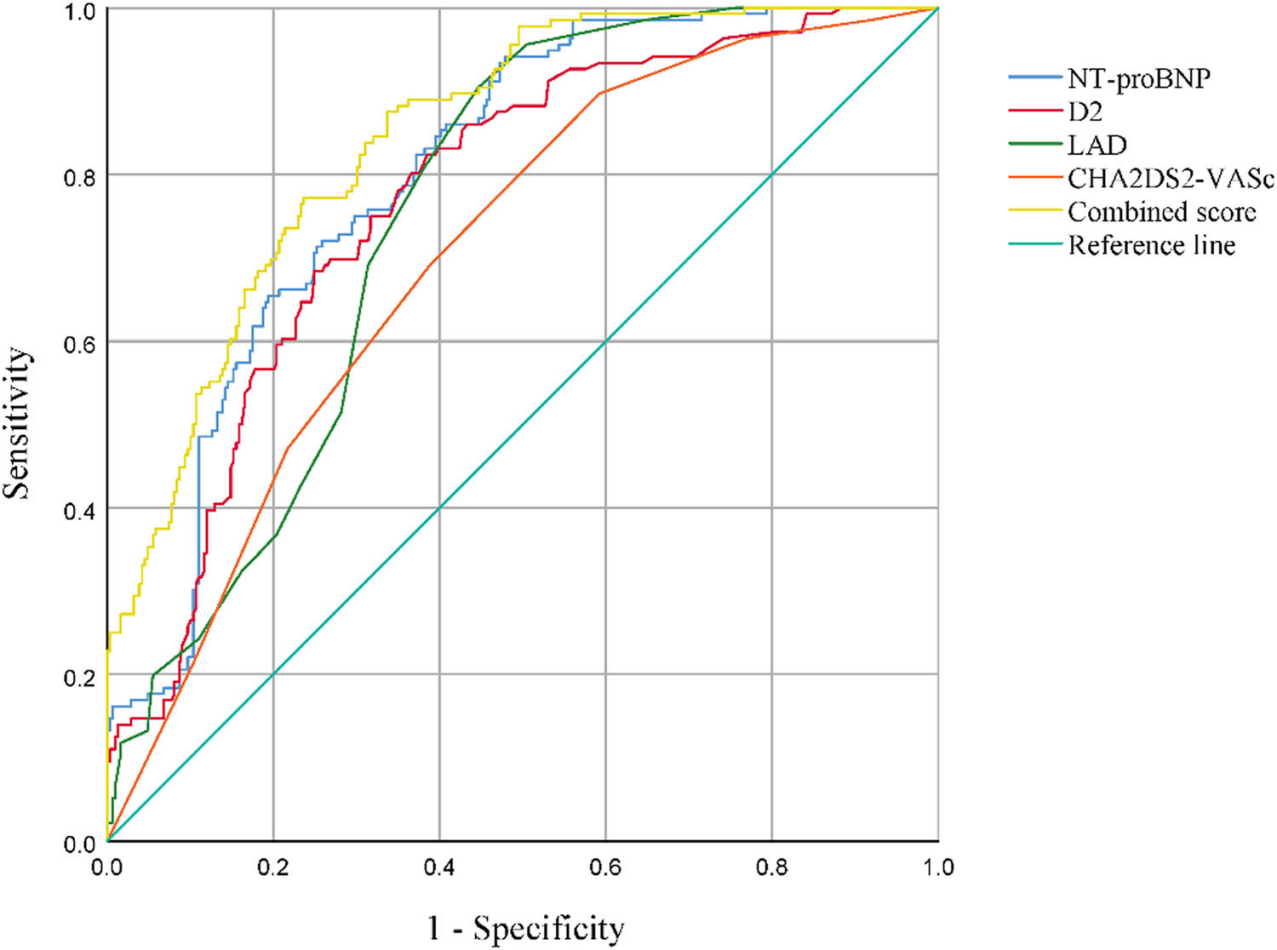
Receiver‐operator characteristic curves for NT‐proBNP, d‐dimer, and LAD to predict NVAF with Stroke. Combined score, NT‐proBNP combined with d‐dimer and LAD. D2, d‐ dimer; LAD, left atrial diameter; NT‐proBNP, N‐terminal pro B‐type natriuretic peptide.

ROC analysis was used to evaluate the predictive value of the risk factors for the occurrence of IS events in patients with NVAF. As shown in Figure 2 and Table 3, NT‐proBNP, d‐dimer, LAD, and CHA2DS2‐VASc scores had a high diagnostic value for the development of IS in patients with NVAF (area under the curve [AUC]: 0.801, 95% CI: 0.76–0.84; AUC: 0.770, 95% CI: 0.72–0.85; AUC: 0.752, 95% CI: 0.71–0.80; AUC: 0.70, 95% CI: 0.65–0.75, *p* < .01). The optimal cutoff value for NT‐ProBNP was 715 pg/ml, with a sensitivity of 72.1% and specificity of 25.9%. The optimal cutoff values for d‐dimer, LAD, and CHA2DS2‐VASc scores were 0.515 ng/ml, 38.5 mm, and 3.5 points, respectively, with sensitivities of 82.4%, 90.4%, and 69.1% and specificities of 61.5%, 55.3%, and 61.2%, respectively. NT‐ProBNP and d‐dimer levels combined with LAD improved the predictive value of a single indicator (AUC: 0.846, 95% CI: 0.81–0.88, *p* < .01) with a sensitivity of 77.2% and a specificity of 76.4%. Compared with the CHA2DS2‐VASc score, the AUC, sensitivity, and specificity of the combined index were higher (0.846 vs. 0.702, 77.2% vs. 69.1%, and 76.4% vs. 61.2%, respectively; *p* < .01).

**Table 3:** Receiver operating characteristic analysis of risk factors

Risk factors	AUC	95% CI	*p* value	Cutoff	Sensitivity (%)	Specificity (%)
NT‐proBNP	0.801	0.76–0.84	<.01	715 pg/ml	72.1	25.9
d‐dimer	0.770	0.72–0.85	<.01	0.515 ng/ml	82.4	61.5
LAD	0.752	0.71–0.80	<.01	38.5 mm	90.4	55.3
CHA2DS2‐VASc	0.702	0.65–0.75	<.01	3.5	69.1	61.2
Combined score^a^	0.846	0.81–0.88	<.01	0.291	77.2	76.4

Abbreviations: AUC, area under the curve; CI, confidence interval; LAD, left atrial diameter; NT‐proBNP, N‐terminal pro B‐type natriuretic peptide.

^a^ NT‐proBNP combined with d‐dimer and LAD.

In addition, the included patients were divided into two groups according to the optimal cutoff values of NT‐proBNP, d‐dimer, and LAD (Tables S2 and S3). The high‐level group (patients in the NT‐proBNP ≥ 715 pg/ml group, d‐dimer ≥ .515 ng/ml group, and LAD ≥ 38.5 mm group) were older, had a higher incidence of CHA2DS2‐VASc score ≥ 2 points (90.4% vs. 77.9%; 92.2% vs. 72.9%; 90.4% vs. 72.3%; *p* < .01), and had a significantly higher risk of IS (72.1% vs. 25.9%; 82.4% vs. 38.5%; 90.4% vs. 44.7%, *p* < .01).

## 4 DISCUSSION

Our research investigated the potential predictive value of NT‐proBNP, d‐dimer, and the echocardiographic parameter LAD for the development of IS in NVAF patients who were at a high risk for cardiogenic stroke. The results showed that NT‐proBNP, d‐dimer, and LAD levels were significantly higher in NVAF patients with stroke than in those without stroke. Increased levels of NT‐proBNP, d‐dimer, and LAD are of great value in confirming the diagnosis of IS and can be used as potential predictors of IS in NVAF patients.

BNP is produced primarily by cardiomyocytes in response to increased end‐diastolic pressure and/or volume expansion and then enzymatically cleaved to NT‐proBNP.^24^, ^25^ Previous research^26^, ^27^, ^28^ have found that NT‐proBNP is a predictor of AF and thromboembolic events and is independently associated with an increased risk of IS. In addition, a single‐center study^29^ showed that the inclusion of NT‐proBNP in the CHA2DS2‐VASc score increased the predictive ability of the risk of IS or systemic thromboembolism by 17% in anticoagulated patients with AF. NT‐proBNP levels were significantly higher in patients with IS than in those without IS, and there was a definite correlation between NT‐proBNP and IS (AUC: 0.801, 95% CI: 0.76–0.84, cutoff point: 715.0 pg/ml). A previous study^30^ found that NT‐proBNP levels 24 h after acute IS were higher than those in controls, which is consistent with our findings.

Among the coagulation indicators in patients with AF, plasma d‐dimer has been extensively studied as an indicator of thrombosis.^31^ Levels of d‐dimer are elevated compared with matched controls in sinus rhythm and appeared to remain elevated despite successful cardioversion, demonstrating a correlation between d‐dimer and NVAF.^15^ In an observational study,^32^ AF patients with high d‐dimer levels also had a higher risk of IS, transient ischemic attack, and arterial thrombotic events. Numerous studies^33^, ^34^, ^35^ had reported that d‐dimer level is elevated during acute stroke, is associated with stroke subtypes and volume, and is significantly elevated in cardioembolic ischemic stroke. In recent years, prospective studies^36^, ^37^ have revealed that d‐dimer levels were correlated to acute stroke, and can be a valuable and independent short‐term prognostic marker for acute stroke. Therefore, assessment of d‐dimer levels is of great value in the early prevention of IS events in patients with NVAF. The incidence of stroke was also significantly higher in the d‐dimer level ≥ 0.515 ng/ml group in our study (82.4% vs. 38.5%, *p* < .01).

A meta‐analysis of 22 clinical studies by Njoku et al.^38^ suggested that the prevalence of AF is significantly and positively correlated with increased LAD, leading to myocardial dysfunction, hemodynamic changes, and inflammatory factors that increase the incidence of thrombosis. Left atrial enlargement may also lead to IS and thrombosis by promoting endothelial damage.^39^, ^40^ In our study, decreased LVEF and increased LVDd were also independent risk factors for the occurrence of IS in patients with NVAF. Therefore, we speculate that LVEF and LVDd could also predict the occurrence of cardiogenic cerebral embolism to a certain extent.

Despite widespread clinical use, there are limitations in the ability of the CHA2DS2‐VASc score to predict the risk of IS in patients with NVAF. Evidence^41^ from a recent systematic review shows that this score has not ideal predictive power (c‐statistic of 0.6–0.7). Our study confirms to some extent the potential predictive value of the 3 identified risk factors or biomarkers (LAD, NT‐proBNP and d‐dimer), which will likely contribute to the refinement of the CHA2DS2‐VASc score and be useful for the early benefit of NVAF patients. The present study also has some limitations. First, this was a single‐center retrospective study with limited sample size; a large multicenter study is required to reduce this bias. Second, considering that this is an observational research, further prospective studies are needed to measure the true value of these indicators.

## 5 CONCLUSION

This study indicates that NT‐proBNP, d‐dimer, and LAD are reliable biomarkers for detecting the occurrence of IS and can be used as independent predictors of IS in patients with NVAF. NT‐proBNP and d‐dimer levels combined with LAD had higher sensitivity and specificity in predicting IS in NVAF than the CHA2DS2‐VASc score. The results of this study will help further screen for NVAF patients at a higher risk of IS and help clinicians administer anticoagulation therapy as soon as possible.

## AUTHOR CONTRIBUTIONS

Zican Shen and Dong Chen analyzed the data, drafted the manuscript, and were the major contributors in writing this manuscript. Hao Cheng and Feng Tan collected data. Wei Fang, Sunan Wang, Jianwei Yan, and Haiming Deng assisted with the research and analysis. Jianbing Zhu conceived and developed the study, provided financial support, and performed analytical revisions and review of the article.

## CONFLICTS OF INTEREST

The authors declare no conflicts of interest.

## Supporting information

Supporting information.

## Data Availability

The datasets used and analyzed in this study are available from the corresponding authors upon reasonable request.
