# Supplementary material for: N‐terminal pro‐B‐type natriuretic peptide and D‐dimer combined with left atrial diameter to predict the risk of ischemic stroke in nonvalvular atrial fibrillation
Source: Clin Cardiol. 2022 Oct 8;46(1):41–8. doi: 10.1002/clc.23933 (PMC9849441; doi:10.1002/clc.23933)
Supplement: Supplementary file 1 — Supporting information. [file CLC-46-41-s001.pdf]

Supplementary Table 1. Risk factors included in the CHA2DS2-VASc score

Risk Factors	CHA2DS2-VASc Score
Congestive heart failure/LV dysfunction	1
Hypertension	1
Age≥75(years)	2
Diabetes mellitus	1
Stroke/TIA/thromboembolism	2
Vascular disease	1
Age 65–74 (years)	1
Sex category (i.e., female sex)	1

Abbreviations: LV dysfunction: Left ventricular dysfunction; TIA: Transient ischemic attack.

Supplementary Table 2: Clinical data of included patients according to the NT-proBNP and D-dimer.

Variable	NT-proBNP (pg/ml)		*P*-value	D-dimer (ng/mL)		*P*-value
	<715 (n=267)	≥715 (n=178)		<0.515 (n=214)	≥0.515 (n=231)	
Age, years (mean ± SD)	70.5(±11.1)	72.5(±10.1)	0.056	67.7(±10.5)	74.7(±9.9)	<0.001
Gender, Male (n, %)	143(53.6)	104(58.4)	0.311	130(60.7)	117(50.6)	0.032
Smoking (n, %)	64(24.0)	42(23.6)	0.928	62(29.0)	44(19.0)	0.014
Hypertension (n, %)	142(53.2)	100(56.2)	0.534	108(50.5)	134(58.0)	0.111
Diabetes mellitus (n, %)	35(13.1)	28(15.7)	0.437	30(14.0)	33(14.3)	0.936
CHD (n, %)	54(20.2)	38(21.3)	0.774	45(21.0)	47(20.3)	0.859
Previous ischemic stroke (n, %)	38(14.2)	40(22.5)	0.025	30(14.0)	48(20.8)	0.061
Ischemic stroke (n, %)	80(25.9)	98(72.1)	<0.01	119(38.5)	112(82.4)	<0.01
D-dimer, ng/mL (median, IQR)	0.4 (0.2-1.0)	0.7 (0.3-1.6)	<0.01			
NT-proBNP, pg/ml (median, IQR)				529.3 (124.1-782.5)	684.0 (382.1-1324.3)	<0.01
LAD, mm (mean ± SD)	37.6(±6.4)	41.8(±5.3)	<0.01	37.3(±6.0)	41.2(±6.1)	<0.01
LVDd, mm (mean ± SD)	44.5(±5.0)	46.7(±6.0)	<0.01	44.1(±5.2)	46.6(±5.5)	<0.01
LVEF, % (mean ± SD)	59.9(±5.5)	56.3(±7.0)	<0.01	60.1(±5.9)	56.9(±6.4)	<0.01
CHA2DS2-VASc score (n, %)			0.007			<0.01
Score＝0	24(9.0)	3(1.7)		22(10.3)	5(2.2)	
Score＝1	35(13.1)	14(7.9)		36(16.8)	13(5.6)	
Score≥2	208(77.9)	161(90.4)		156(72.9)	213(92.2)	

Abbreviations: SD, standard deviation; IQR, inter quartile range; CHD, coronary heart disease; NT-proBNP, N-terminal pro B-type natriuretic peptide; LAD, left atrial diameter; LVDd, left ventricular end-diastolic dimension; LVEF, left ventricular

ejection fraction.

Supplementary Table 3: Clinical data of included patients according to the LAD.

Variable	LAD (mm)		*P*-value
	<38.5<br>(n=184)	≥38.5<br>(n=261)	
Age, years (mean ± SD)	69.8(±11.5)	72.4(±10.1)	0.017
Gender, Male (n, %)	101(54.9)	146(55.9)	0.827
Smoking (n, %)	50(27.2)	56(21.5)	0.163
Hypertension (n, %)	91(49.5)	151(57.9)	0.080
Diabetes mellitus (n, %)	20(10.9)	43(16.5)	0.095
CHD (n, %)	38(20.7)	54(20.7)	0.992
Previous ischemic stroke (n, %)	25(13.6)	53(20.3)	0.066
Ischemic stroke (n, %)	138(44.7)	123(90.4)	<0.01
D-dimer, ng/mL (median, IQR)	0.4(0.2-0.8)	0.8(0.3-1.5)	<0.01
NT-proBNP, pg/ml (median, IQR)	334.5<br>(67.3-698.8)	728.0<br>(500.5-1355.0)	<0.01
LAD, mm (mean ± SD)			
LVDd, mm (mean ± SD)	43.4(±4.9)	46.8(±5.5)	<0.01
LVEF, % (mean ± SD)	60.0(±6.1)	57.3(±6.4)	<0.01
CHA2DS2-VASc score (n, %)			<0.01
Score＝0	19(10.3)	8(3.1)	
Score＝1	32(17.4)	17(6.5)	
Score≥2	133(72.3)	236(90.4)	
